# Capsule Endoscopy: Current Trends, Technological Advancements, and Future Perspectives in Gastrointestinal Diagnostics

**DOI:** 10.3390/bioengineering12060613

**Published:** 2025-06-04

**Authors:** Chang-Chao Su, Chu-Kuang Chou, Arvind Mukundan, Riya Karmakar, Binusha Fathima Sanbatcha, Chien-Wei Huang, Wei-Chun Weng, Hsiang-Chen Wang

**Affiliations:** 1 Division of Gastroenterology and Hepatology, Department of Internal Medicine, Ditmanson Medical Foundation Chia-Yi Christian Hospital, Chia-Yi 60002, Taiwan; 06155@cych.org.tw (C.-C.S.); vacinu@gmail.com (C.-K.C.); 2 Obesity Center, Ditmanson Medical Foundation Chia-Yi Christian Hospital, Chia-Yi 60002, Taiwan; 3 Department of Medical Quality, Ditmanson Medical Foundation Chia-Yi Christian Hospital, Chia-Yi 60002, Taiwan; 4 Department of Mechanical Engineering, National Chung Cheng University, 168, University Rd., Min Hsiung, Chia-Yi 62102, Taiwan; d09420003@ccu.edu.tw (A.M.); karmakarriya345@gmail.com (R.K.); 5 Department of Biomedical Imaging, Chennai Institute of Technology, Sarathy Nagar, Chennai 600069, India; 6 Department of Biotechnology, Karpagam Academy of Higher Education, Salem-Kochi Hwy, Coimbatore 641021, India; binushafathimasanbasha14@gmail.com; 7 Department of Gastroenterology, Kaohsiung Armed Forces General Hospital, 2, Zhongzheng 1st.Rd., Lingya District, Kaohsiung City 80284, Taiwan; forevershiningfy@yahoo.com.tw; 8 Department of Nursing, Tajen University, 20, Weixin Rd., Yanpu Township, Pingtung County 90741, Taiwan; 9 Department of Medical Research, Dalin Tzu Chi Hospital, Buddhist Tzu Chi Medical Foundation, No. 2, Minsheng Road, Dalin, Chia-Yi 62247, Taiwan; 10 Hitspectra Intelligent Technology Co., Ltd., Kaohsiung 80661, Taiwan

**Keywords:** capsule endoscope, narrow-band imaging, white-light imaging, machine learning, steerable capsule endoscope, magnetic capsule endoscopy, robotic capsule endoscopy, hybrid capsule endoscopy, PillCam

## Abstract

Capsule endoscopy (CE) has revolutionized gastrointestinal (GI) diagnostics by providing a non-invasive, patient-centered approach to observing the digestive tract. Conceived in 2000 by Gavriel Iddan, CE employs a diminutive, ingestible capsule containing a high-resolution camera, LED lighting, and a power supply. It specializes in visualizing the small intestine, a region frequently unreachable by conventional endoscopy. CE helps detect and monitor disorders, such as unexplained gastrointestinal bleeding, Crohn’s disease, and cancer, while presenting a lower procedural risk than conventional endoscopy. Contrary to conventional techniques that necessitate anesthesia, CE reduces patient discomfort and complications. Nonetheless, its constraints, specifically the incapacity to conduct biopsies or therapeutic procedures, have spurred technical advancements. Five primary types of capsule endoscopes have emerged: steerable, magnetic, robotic, tethered, and hybrid. Their performance varies substantially. For example, the image sizes vary from 256 × 256 to 640 × 480 pixels, the fields of view (FOV) range from 140° to 360°, the battery life is between 8 and 15 h, and the frame rates fluctuate from 2 to 35 frames per second, contingent upon motion-adaptive capture. This study addresses a significant gap by methodically evaluating CE platforms, outlining their clinical preparedness, and examining the underexploited potential of artificial intelligence in improving diagnostic precision. Through the examination of technical requirements and clinical integration, we highlight the progress made in overcoming existing CE constraints and outline prospective developments for next-generation GI diagnostics.

## 1. Introduction

A German physician, Bozini, invented an instrument called Lichtleiter, which is used to examine the bladder, rectum, vagina, and upper respiratory tract. His invention is considered the first step in the development of the endoscope [1]. In 1853, Antonin Desormeaux developed an open-tube endoscope for the examination of the urethra and bladder [2]. Due to the complexity of the large intestine’s shape, it is challenging to insert an endoscope into it. Consequently, numerous endoscopes have been developed to facilitate entry into the large intestine [3]. The second major step in endoscopy is capsule endoscopy (CE), an ingestible wireless camera that captures images of the gastrointestinal mucosa [4]. It was developed in 2001 by Gavriel Iddan [5]. CE consists of three parts: a computer with software, a data recorder, and a capsule. The capsule has a camera that is ingested into the patient’s body, and a data recorder is placed on the patient’s body with sensor pads. A computer with software is used for analyzing images from the data recorder. A capsule features a camera comprising an optical dome, a light source, a CMOS sensor, a battery, a lens, and a wireless transmitter. The capsule captures two images per second and records approximately 50,000 images during an 8-h examination [6]. The first test was conducted on pigs, and human trials commenced in 2001 [7,8]. The patients do not require sedation for the CE examination. This capsule can examine the GI tract from the esophagus to the small intestine, a region that cannot be examined by traditional endoscopy [9]. By consuming a CE, we can detect ulcers and bleeding in the gastrointestinal tract, which can be visualized via wireless video transmission and treated by surgeons [10]. CE is the examination tool for Crohn’s disease, and it can aid in diagnosing diseases such as inflammatory bowel disease [11]. The ultra-wideband (UWB) technique is used to transmit images from an on-body device. It enables high data transfer rates with low power consumption and features a large bandwidth that supports high-resolution images [12]. The first video capsule, M2A, uses four LEDs as its light source. The images are captured every 0.5 s by a CMOS chip and then transmitted at a radio frequency. The battery life is 6 h, and it features a 140-degree view. The CMOS chip is composed of 256 × 256 pixels [13].

Many studies have shown that CE can diagnose the early stages of Crohn’s disease more effectively than colonoscopy and radiology techniques [14]. CE is a less invasive method that reduces patient discomfort and allows easy access to previously inaccessible structures, such as the small intestine [15]. The patient should not eat for 12 h before the examination. Patients should wear a bag containing storage for the device. Before swallowing the capsule, eight skin antennas, similar to ECG electrodes, are attached to the anterior abdominal wall of the patient [16]. For small bowel treatment, the bowel should be cleansed with 2 L of polyethylene glycol solution (PEG) 16 h prior to ingesting the capsule. Patients can drink liquid after 2 h and eat food after 4 h following capsule ingestion [17,18]. Patients are instructed to fast for 5 h before the examination. The patients swallow the capsule using a standardized ECE ingestion protocol. The patient should ingest the capsule by drinking 10 mL of water while in a sleeping position. After ingesting the capsule, the patient should remain in position for 2 min and then be raised by a nurse to 30 degrees for 2 min, 60 degrees for 1 min, and then to a sitting position for 1 min. The patient should not speak during this process [19]. A total of 3 L of PEG osmotic solution is used to clean the bowel before and on the day of the CE examination, and a booster dose of sodium phosphate is added to improve the capsule’s movement [20,21]. A capsule-loading device is available for patients who are unable to swallow the capsule. The device is inserted into the endoscope’s working channel, and the capsule is placed in a cup at the tip of the endoscope. The capsule is then released into the duodenum with the help of the endoscope’s working channel [22]. Many swallowable capsules have been developed in recent decades to examine the GI tract [23]. More than 600,000 capsules have been used worldwide in clinical practices [24].

## 2. Comparison Between Traditional and Capsule Endoscopy

Traditional endoscopy comprises a long flexible tube that is inserted into the patient’s mouth, rectum, urethra, or vagina. This method requires sedation to comfort the patient because the process can be painful and uncomfortable. CE is a capsule-shaped instrument that involves swallowing a pill that travels through the gastrointestinal tract. It is a painless and minimally invasive method [25,26,27,28]. CE can examine the GI tract without causing pain or requiring sedation, as examining the small intestine can be challenging. Traditional endoscopy can reach the stomach and colon but cannot access the small intestine [29]. CE is very comfortable for patients, as it is easy and safe to perform, and it makes gastrointestinal regions visible. In contrast, traditional endoscopy can cause bleeding, perforation, and infection [30,31]. CE is designed to be non-invasive, whereas traditional endoscopy is minimally invasive [32,33]. During an 8-h examination, CE produces 50,000 images, and it takes approximately 2 h for doctors to review the image. In contrast, traditional endoscopy lasts around 30 min and captures only 30 to 50 images [34,35,36]. The limitations of CE include its inability to take biopsies or perform therapeutic procedures. In contrast, traditional endoscopy allows for the collection of tissue samples from patients and enables the diagnosis and treatment of various conditions. Biopsies taken from patients help to diagnose diseases of specific organs [37,38,39]. Endoscopy can diagnose and treat inflammatory bowel disease, but colonoscopy can only evaluate inflammatory bowel diseases [40,41]. Narrow-band imaging is utilized in traditional endoscopy to enhance image quality and improve visualization of mucosal surface architecture; however, in CE, NBI technology has not been widely adopted, which is a major drawback. This narrow-band imaging utilizes a specific wavelength to highlight differences in tissue structure, which helps to identify abnormalities more easily [42]. CE is more comfortable for the patients compared to traditional wired endoscopy. The process of swallowing a capsule is much easier, not unlike the insertion of a flexible tube, which can be very uncomfortable [43]. Precise localization of the capsule inside the gastrointestinal tract is crucial for determining the anatomical position of lesions and strategizing subsequent procedures. A variety of strategies have been explored to address this challenge. Magnetic CE systems often utilize external magnetic fields and sensors to track the capsule’s position in real time. Robotic and hybrid capsules may incorporate magnetic localization alongside inertial measurement units (IMUs), accelerometers, and gyroscopes to enhance spatial tracking. Image-based methodologies, including the comparison of anatomical landmarks and the application of AI-driven frame classification, have been investigated. Nonetheless, attaining high-precision localization in the small intestine is challenging due to its length and peristaltic motion. The future advancement of multimodal tracking systems integrating electromagnetic and vision-based data may substantially improve diagnostic accuracy and facilitate targeted therapies.

**Figure 1:**
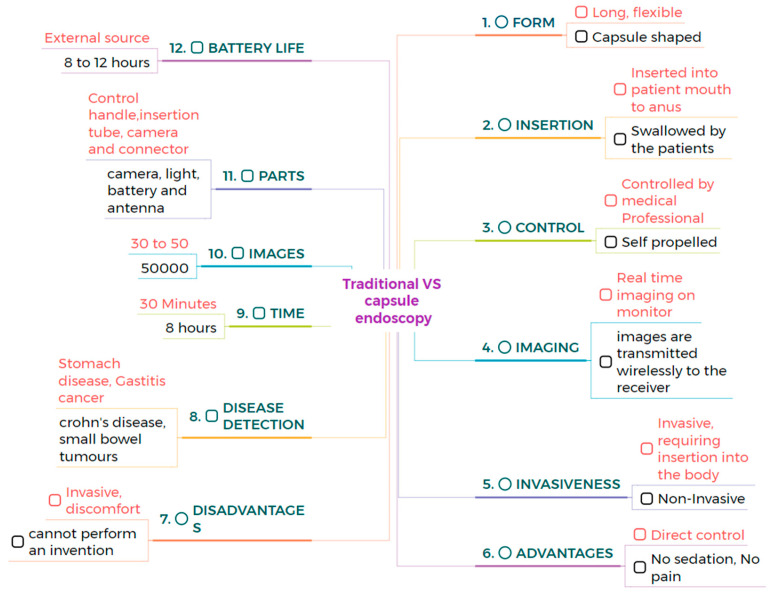
The direct comparison between the traditional and the capsule endoscope.

The average size of CE measures 32 mm in length and 12 mm in diameter. The MicroCam capsule has a length and diameter of 24.5 × 10.8 mm, while the OMOM capsule is slightly larger at 25.4 mm and has a diameter of 11 mm. The PillCam measures a length of 26.2 mm and a diameter of 11.4 mm, and the endocapsule has a length of 26 mm and a diameter of 11 mm. Traditional endoscopy devices are larger. The Olympus endoscopy measures a length of 2 m and has a diameter of 9.2 mm. These variations highlight the significant size difference between capsule endoscopy devices and traditional endoscopes. Capsule endoscopy is very comfortable compared to traditional endoscopy, enabling less invasive options for examining the gastrointestinal tract [44,45]. Traditional endoscopy can be operated manually, with robotic assistance, or connected to an assistance system, while CE is a self-propelled method. This makes CE more convenient and less invasive, as it does not require manual operation or any systems to move the capsule [46,47]. CE examines different regions of the GI tract with minimal discomfort. CE is a self-propelled method that eliminates the need for manual operation or robotic assistance [48]. CE comprises a camera, LED, antenna, light, and battery, and the outer material of CE is made up of biocompatible polycarbonate. This construction enables CE to navigate the gastroin-testinal tract safely and non-invasively, whereas traditional endoscopy consists of a control handle, insertion tube, camera, and connector. These devices are constructed from flexible polyurethane coated with a hydrophilic material, which enhances ma-neuverability within the digestive tract [49,50,51]. CE has an average battery life of 8 to 12 h and an average weight of approximately 3.5 g. These variations in battery and weight among specific models highlight the differences between them. For example, PillCam SB2 has a lifespan of 8 h and a weight of 2.8 g. It is lightweight and suitable for use in examinations. The endocapsule has a similar battery life to the PillCam SB2 but weighs 3 g. In contrast, the MicroCam offers an extended battery life of 11 h and weighs only 3 g, providing advantages for longer examinations. The PillCam ESO2 capsule lasts for 8 h on a single charge, weighing 4 g, while the PillCam SB2EX boasts the longest battery life of 12 h and weighs 2.8 g. This allows for uninterrupted power and transmission throughout the procedure [52]. CE can detect small bowel tumors and Crohn’s disease, offering a non-invasive diagnostic tool for these conditions, similar to traditional endoscopy, which can detect stomach diseases and gastric cancers. Both methods are valuable because they target different areas of the gastrointestinal tract [53,54,55]. PillCam SB3 provides a resolution of 340 × 340. In contrast, the endocapsule offers a higher resolution of 512 × 512. PillCam SB2EX and PillCam ESO2 both provide the same resolution of 256 × 256. In contrast, the MicroCam offers a resolution of 320 × 320, while the OMOM capsule boasts a resolution of 640 × 480, surpassing that of other capsules. However, Olympus endoscopy offers a resolution of 350 × 360 [56,57]. The comparison between traditional and capsule endoscopes is shown in Figure 1. However, due to significant variability in clinical study designs, disease-specific endpoints, and diagnostic criteria across the literature, a standardized tabular comparison of sensitivity, specificity, and diagnostic accuracy between capsule and traditional endoscopy was not feasible. 

## 3. Types of Endoscopes

### 3.1. Steerable Capsule Endoscope

**Figure 2:**
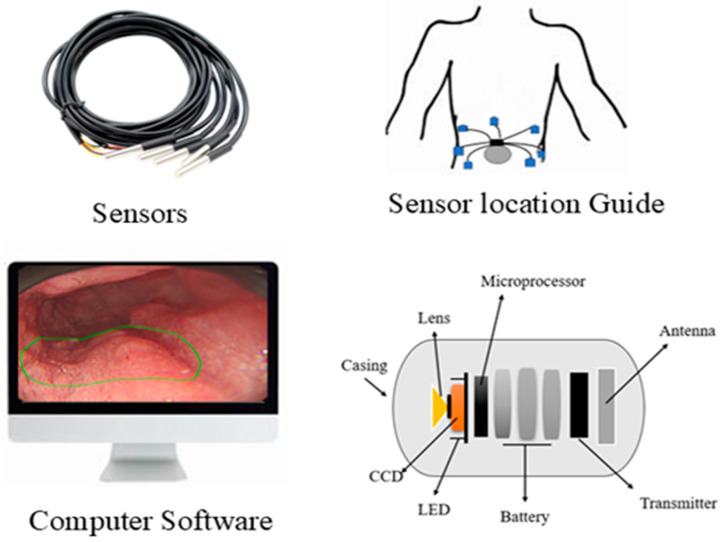
Working principle of the steerable capsule endoscope.

The steerable capsule endoscope was invented by Carta et al. and features a 3D steerable locomotion system (size: 30 cm long and 15 cm wide), as shown in Figure 2. Most capsules are not manufactured with active locomotion; however, this is sufficient for small bowel analysis. The capsule should have the ability to steer for the examination of GI tract sections, particularly in the stomach. With the help of active locomotion, the capsule can accelerate, decelerate, and come to a stop [58]. The capsule tissue interaction force is measured by a flexible force sensor. The flexible force sensor consists of eight force-sensitive elements surrounded by an internal permanent magnet (IPM) [59]. 

### 3.2. Magnetic Capsule Endoscopy

**Figure 3:**
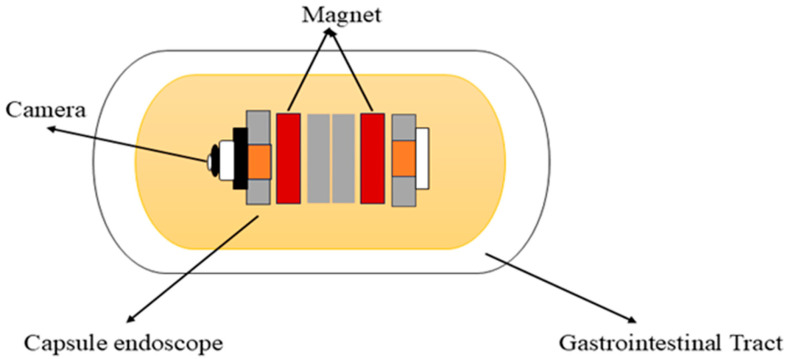
Working principle of the magnetic capsule endoscope.

Carpi et al. (2006) proposed the concept of a magnetically controlled capsule for observing the gastric cavity, which was developed in a telerobotics lab at the University of Utah [51,60]. This system comprises an endoscopic capsule with a permanent magnet embedded in the body, a control system, and eight air-cored electromagnetic coils, as shown in Figure 3. It is designed to perform 5-DOF motion, including 3-DOF translational motion and 2-DOF rotational motion. This enables orientation-independent driving (OID) control of the capsule [61]. This advancement in magnetic capsule endoscopy improves examination efficiency, accuracy, and navigation in the gastrointestinal tract. To attain full visualization of the upper gastrointestinal mucosa, a multiplanar reconstruction CT modeling technique is employed to ascertain the optimal placement of the magnetic capsule endoscope within the upper gastrointestinal tract and identify the most suitable position for a manual magnet to facilitate its passage through the pylorus [62]. It is used for various purposes in the diagnostics and treatments of the gastrointestinal tract. It consists of cameras, batteries, LEDs, and antennas for accurate localization within the gastrointestinal tract. Additionally, it can also detect gastrointestinal lesions, including bleeding, and identify melena, hematochezia, and iron deficiency anemia of unclear origin [63]. It can deliver drugs to specific areas in the digestive tract by using a dual-function permanent magnet for both movement and injection activation. The dimensions of this capsule are 12.5 mm in diameter and 34.6 mm in length [64]. Magnetic capsule endoscopy has some limitations, such as low-quality images, incomplete analysis of the esophagus, and restricted control methods [65].

### 3.3. Robotic Capsule Endoscopy 

**Figure 4:**
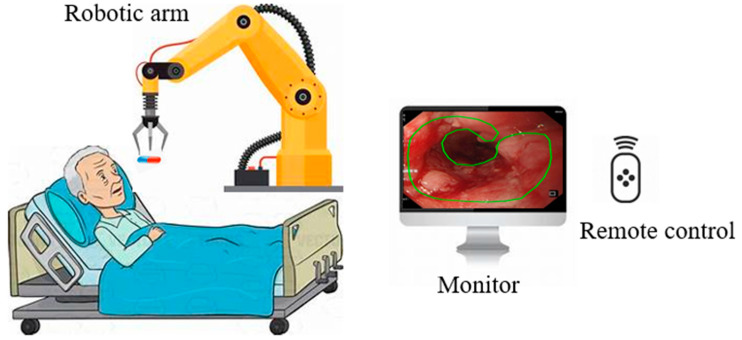
Robotic capsule endoscope.

Based on medical procedures in the stomach, Yim et al. proposed a magnetic robot capsule endoscopy [66]. It utilizes magnetic fields to control the movement and function of the capsule within the gastrointestinal tract, as illustrated in Figure 4 [67]. This technology plays a crucial role in the identification of various pathologies, such as tumors, gastrointestinal bleeding, Crohn’s disease, and celiac disease [68]. The robotic capsule consists of six modules: (1) locomotion, (2) telemetry, (3) localization, (4) visualization, (5) powering, and (6) diagnosing and treatment tools [69]. Robotic capsules can also deliver drugs to the patient [70]. These systems integrate with small permanent magnets within the capsule, and the magnets are controlled by magnetic fields generated by an external magnet or coils. [71,72]. Robotic capsule endoscopy is used for examining the digestive system, primarily for diagnosing gastrointestinal tissue irregularities, particularly in the small intestine, without the need for sedation [73]. Microcontrollers, micro propellers, wireless communication modules, and advanced sensors are all integrated into these robots [74]. One main limitation of the robotic wireless capsule endoscopy is that it cannot detect and treat lesions autonomously.

### 3.4. Hybrid Capsule Endoscopy

**Figure 5:**
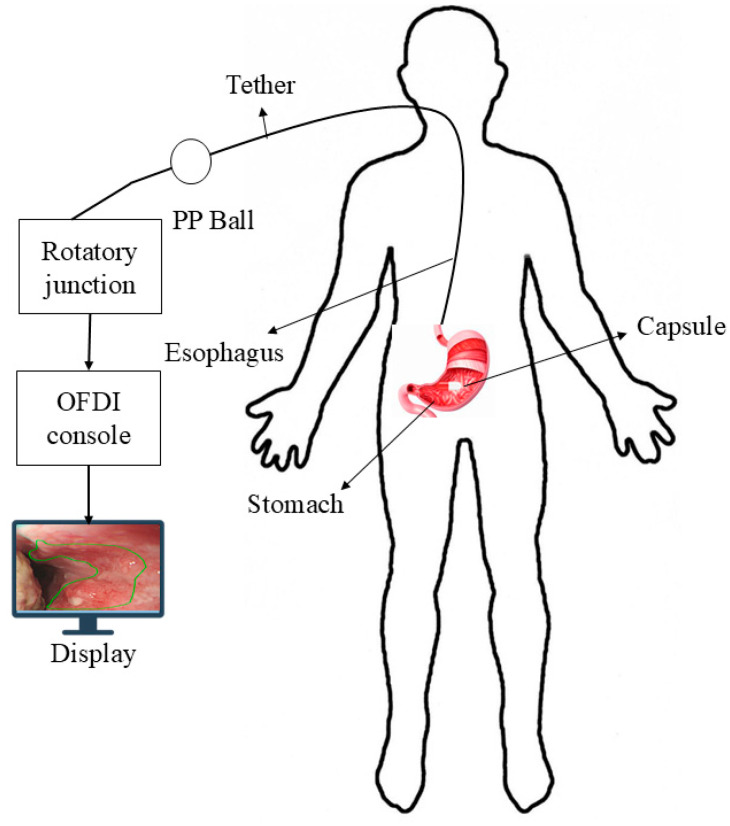
Hybrid capsule endoscope.

A hybrid wireless capsule has been developed by Simi et al. for evaluating the digestive tract (Figure 5). With active hybrid locomotion, they created a micro robot in the shape of a capsule, utilizing a combination of external magnetic dragging and an internal actuation mechanism. External locomotion is achieved by using a magnet inside the capsule coupled with an external magnetic field generated by coils or permanent magnets. The internal actuator uses small permanent magnets that interact with an external magnetic field [75]. Together with Tianjin University of Technology and Kagawa University, a magnetic capsule was created that utilizes a hybrid motion, merging fish-like locomotion with spiral motion. It utilizes a gravity method and generates a fin movement by employing an axial magnet. It also utilizes a radial magnet to control the rotation of the spiral body. The capsule robot measures 90 mm in length and has a diameter of 10 mm. A comparison between different types of capsules is shown in Table 1.

## 4. Current State of the Art

### 4.1. Current Technologies

**Figure 6:**
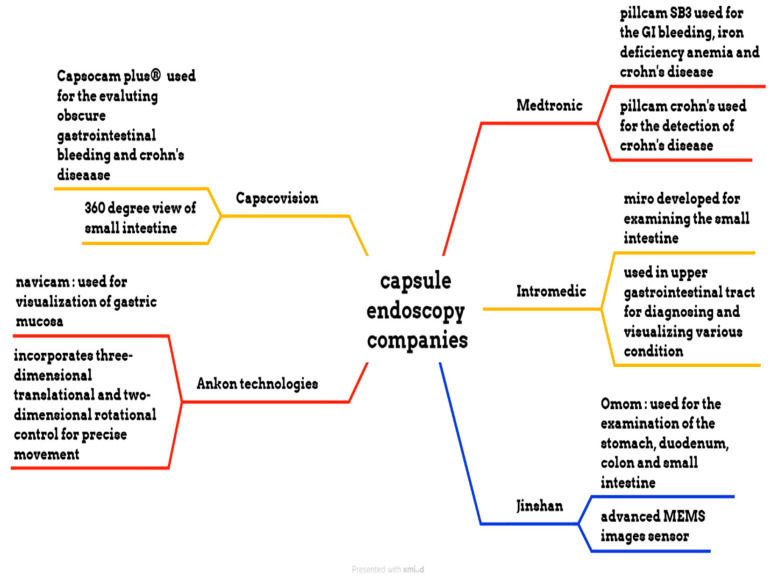
Different endoscopic companies’ capsules.

Medtronic is a medical device manufacturing company that manufactures the PillCam SB 3 capsule, the PillCam Crohn’s capsule, the PillCam COLON 2 capsule, and the PillCam patency capsule, as shown in Figure 6. PillCam SB 3 is used for the diagnosis of GI bleeding, iron deficiency anemia, and Crohn’s disease. It has complete visualization of the small bowel through a wide-angle view and adaptive frame rate. It captures two to six images per second, based on the movement of the capsule, and features excellent image quality and exclusive software features. The PillCam Crohn’s capsule is used for the detection of Crohn’s disease. It has a 172° field of view and does not require sedation or radiation. It captures 4 to 35 images per second, depending on the capsule speed. The PillCam COLON 2 capsule is utilized for polyp detection. It consists of two camera heads that have an extra-wide angle, providing a 172° view [76,77,78,79]. IntroMedic developed a capsule endoscopy named Miro. This capsule was primarily developed to examine the small intestine, as conventional endoscopy cannot reach it. It captures 320 × 320 pixels and three images per second. This capsule has a battery life of 11 h and weighs 3.3 g [80]. The OMOM capsule is manufactured by Jinshan. It has a 10-h battery life and is used for evaluating the stomach, duodenum, colon, and small intestine. The weight of this capsule is 4.5 g, and its size is 25.4 × 11 mm. It has an advanced MEMS image sensor. The Capsocam Plus^®^ capsule endoscopy is developed by Capsovision. This capsule provides a 360-degree view of the small intestine and generates a higher-quality image. It can evaluate gastrointestinal bleeding and Crohn’s disease. It has a battery life of 15 h [81,82]. The Navicam capsule endoscopy is developed by Ankon Technologies. It features a 140° view and a battery life of 12 h. The length of the capsule is 27 mm, and the diameter is 11.8 mm. The weight of this capsule is 5 g. The image resolution of this capsule is 640 × 480. It is used for visualizing the gastric mucosa. The system uses three-dimensional translational and two-dimensional rotational control to guide the movement of the capsule inside the stomach [83].

### 4.2. Machine Learning Applications of Capsule Endoscopy

For the classification of GI organs using a dataset of 37,307 images from 24 CE videos and 39,781 CE images from 30 videos, Chung et al. (2023) utilized PillCam SB3 for image capture. This dataset was analyzed using Neuro-T, achieving an accuracy, recall, precision, and F1-score of 98%, 97%, 89%, and 92%, respectively. The average accuracy for different organs, such as the stomach, esophagus, colon, and small bowel, is 96%, 98%, 87%, and 8%, respectively. This indicates a high level of precision and reliability in organ classification [84]. In 2023, Park et al. utilized PillCam SB3 in an investigation. They applied the Gaussian filter model for the classification of organs, such as the stomach, colon, and small bowel, using 23,959,32 images from 126 patients, and obtained accuracies of 95% in each organ, as well as an overall accuracy and F1-score of 97.1% [85]. For the detection of small bowel lesions, Afonso et al. (2021) utilized 21,320 CE images from 2565 patients using PillCam SB3. They achieved an accuracy and specificity of 97.1% and a sensitivity of 95.9% using the Xception model [86]. For detecting ulcers in Crohn’s disease, Klang et al. (2020) utilized 17,640 images obtained with PillCam SB3, and the results indicated an accuracy ranging from 95.4% to 96.7% using a state-of-the-art Xception model [87]. Aoki et al. (2020) utilized 6,503 images and blood samples from 29 patients, as well as 21,344 images of the small bowel from 10 patients, using PillCam SB3. For the detection of blood using the ResNet model, an accuracy of 99.89%, a sensitivity of 96.63%, and a specificity of 99.96% were achieved [88]. Chul Chung et al. (2020) utilized a dataset comprising 7,556 images from 526 SBCE videos, which were obtained using PillCam SB3. They classified them into two categories: hemorrhagic lesions (red spots, angioectasia, and active bleeding) and ulcerative lesions (erosions, ulcers, and strictures). They utilized VGG-Net for classification and achieved accuracies of 97.48% for red spots, 99.77% for active bleeding, 97.15% for erosions, 96.86% for ulcers, and 97.58% for strictures, as well as 100% for angioectasia [89]. In 2021, Rustam et al. utilized a dataset comprising 1650 CE images acquired via PillCam SB3 for the classification of bleeding images. They implemented a bleed image recognizer in conjunction with MobileNet, achieving an accuracy of 99%, a precision of 100%, a recall of 99%, an F1-score of 99%, a Cohen’s kappa of 95%, and an accuracy of 97% for the BIR model [90].

In 2021, Mamun et al. conducted a study focused on classifying bleeding and non-bleeding conditions using PillCam SB, generating 2393 images. They employed a fuzzy logic edge detection technique to achieve an accuracy of 98.4%, a sensitivity of 98%, a precision of 93%, and an F1-score of 99% [91]. Yuan et al. (2017) used PillCam SB for the recognition of polyps in CE images. The dataset comprises 3000 normal images and 1,000 polyp images from 35 patients, with the normal images categorized into three types: bubbles, turbid images, and clear images. They utilized a stacked sparse autoencoder with an image manifold constraint (SSAEIM) model for polyp recognition, achieving an accuracy of 98% for WCE images and 99.50%, 99.00%, and 95.50% for bubbles, turbid images, and clear images, respectively [92]. Sindhu et al. (2017) utilized a dataset containing 435 WCE images, which consisted of 187 polyps, 100 tumors, and normal images taken with PillCam SB. They applied a neural network classifier for the automated detection of colonic polyps and tumors, achieving an accuracy of 97.5% for both [93].

Nadimi et al. (2020) utilized 11,300 CE images generated by PillCam COLON 2 for the autonomous detection of colorectal polyps. By using the ZF-Net model, they achieved an accuracy of 98.0%, a sensitivity of 98.1%, and a specificity of 96.3% [94]. Saraiva et al. (2021) employed a TensorFlow and Keras model for the detection of colonic protuberant lesions using 765 images generated by PillCam COLON 2, achieving an accuracy of 97.1%, a sensitivity of 95.4%, and a specificity of 97.3% [95]. Gilabert et al. (2022) utilized 18 videos generated by PillCam COLON 2 for polyp detection, achieving an accuracy of 80.00% and a sensitivity of 87.00% using the RAPID model [96].

Tsuboi et al. (2020) applied a single-shot multi-box detector model for detecting small bowel angioectasia using a dataset of 2237 CE images generated by PillCam SB2. They obtained an accuracy of 90.8%, a sensitivity of 90.9%, and a specificity of 88.2% [97]. Saito et al. (2020) utilized a dataset comprising 17,507 images from 73 patients who underwent PillCam SB2 examinations to automatically classify and detect protruding lesions. They applied a single-shot multi-box detector and achieved a sensitivity and specificity of 90.7% and 79.8% for protruding lesions. Additionally, they achieved sensitivities of 86.5%, 92.0%, 95.8%, 77.0%, and 94.4% for polyps, nodules, epithelial tumors, submucosal tumors, and venous structures, respectively [98]. 

Majtner et al. (2021) utilized a dataset of 7744 images from 38 patients, obtained using PillCam Chron’s capsule, for the autonomous detection and classification of Crohn’s disease lesions in the small bowel and colon. They achieved an accuracy of 98.5% for the small bowel and 98.1% for the colon using the ResNet-50 model [99]. 

Xia et al. (2021) utilized 1,023,955 CE images from 797 patients acquired with Navicam for the detection of gastric focal lesions. They employed the ResNet model for classification, achieving an accuracy of 77.1%, a sensitivity of 96.2%, and a specificity of 76.2% [100]. In 2019, Wang et al. used Navicam for the automatic detection of ulcers from 1504 patients, employing a state-of-the-art detection model and achieving an accuracy of 90.10%, a sensitivity of 90.48%, and a specificity of 90.48%, respectively [101]. 

Bajhaiya et al. (2024) applied the GI net model for the detection and localization of gastrointestinal diseases utilizing a dataset of 3658 CE images, achieving accuracies of 99%, 99.6%, and 99.86% for sensitivity, specificity, and overall accuracy, respectively [102]. Xu et al. (2018) utilized 14,408 images from patients, including 408 normal images and 14,000 abnormal images, for the automatic recognition of polyps. They applied AlexNet for classification and achieved accuracy, sensitivity, and specificity of 99.98%, 99.40%, and 99.93%, respectively [103]. In 2021, Sunitha et al. utilized a dataset containing 420 images to detect bleeding images, applying the AlexNet architecture and achieving an accuracy of 94.5%, a sensitivity of 95.24%, and a specificity of 96.72% [104]. Yuan et al. (2015) applied the SVM model for bleeding frame detection in WCE images. They utilized a dataset consisting of 800 images, including 400 normal and 400 bleeding images, and achieved an accuracy of 95.89%, a sensitivity of 98.77%, and a specificity of 93.45% [105]. Seebutda et al. (2023) employed 48 photos for the segmentation of bleeding regions using the K-means clustering model, attaining accuracy, sensitivity, and precision rates of 84.26%, 69.84%, and 65.70%, respectively [106].

## 5. Discussion and Conclusions

This study analyzed the progression of CE technologies, emphasizing the design, performance metrics, and diagnostic efficacy of steerable, magnetic, robotic, tethered, and hybrid capsule variants. We emphasized differences in resolution (256 × 256 to 640 × 480 pixels), field of view (140°–360°), battery life (8–15 h), and frame rates (2–35 fps) through comparative study, all of which influence diagnostic efficacy. These enhancements represent a substantial progression in the downsizing and efficacy of CE for GI imaging. Notwithstanding these gains, significant limits persist. Many capsule systems continue to be deficient in real-time control, tissue biopsy capabilities, and therapeutic functionalities. Moreover, inadequate intestinal preparation, prolonged transit time, and restricted localization accuracy can compromise imaging quality and diagnostic accuracy. Although robotic and hybrid capsules present intriguing solutions through active locomotion and magnetic steering, their clinical translation is hindered by complexity, expense, and regulatory challenges. The integration of AI into customer experience operations is a significantly underutilized yet high-potential domain. Deep learning models have exhibited remarkable efficacy in identifying bleeding, ulcers, and polyps, achieving classification accuracies over 95% in certain experiments. Nevertheless, these models have not been extensively adopted in clinical environments, primarily due to the limited availability of datasets, challenges in standardization, and the absence of real-time inference capabilities. Future investigations should prioritize:

- Advancement of biopsy-capable and therapeutic capsule designs;
- Improvement of real-time navigation and localization systems;
- Extended-range wireless communication and energy-efficient designs;
- Standardization of datasets to facilitate robust AI model training;
- Clinical trials testing AI-assisted CE diagnostics for regulatory approval.

By integrating engineering advancements with AI-driven software solutions, CE is set to evolve from a passive imaging device to an intelligent, autonomous diagnostic instrument that can revolutionize patient care in gastroenterology. Notwithstanding significant progress, certain critical barriers hinder the extensive clinical application of novel capsule varieties. Elevated manufacturing expenses and technical intricacy—particularly for motorized, steerable, or therapeutic capsules—impede large-scale production. The miniaturization of components without sacrificing functionality continues to pose challenges. Regulatory obstacles, such as protracted FDA approval procedures, additionally impede market access. Moreover, conflicting payment rules and insufficient integration with current healthcare workflows impede implementation. Clinicians may encounter a learning curve with emerging technologies. Overcoming these obstacles via efficient approval processes, economical design, and rigorous clinical validation is crucial for integrating capsule advances into standard GI treatment. Every modern capsule technology encounters specific constraints in clinical applications. Steerable capsules offer directional control but frequently encounter mobility challenges in intricate gastrointestinal geometries, necessitating precise external manipulation. Magnetic capsules rely on substantial external magnetic systems and may encounter localization drift, particularly in deep intestinal areas. Robotic capsules are constrained by power consumption and miniaturization trade-offs, leading to reduced battery life and more cumbersome designs. Tethered capsules offer uninterrupted power and immediate control, although they diminish patient comfort and are less effective for deep intestinal imaging. Moreover, issues influencing patient compliance, such as challenges in swallowing larger pills or inadequate gastrointestinal preparation, can adversely impact diagnostic yield and image quality. CE continues to evolve as a minimally invasive tool for gastrointestinal diagnostics. With innovations in locomotion, image quality, and AI integration, CE is positioned to become more autonomous and clinically impactful. Addressing current technical and clinical challenges will be key to realizing its full potential in routine practice. Innovative innovations in capsule endoscopy are anticipated to greatly enhance patient care in clinical environments. The miniaturization of motors and improved battery efficiency will provide prolonged and more precise inspections, especially in intricate areas such as the small intestine. The incorporation of AI will reduce diagnostic effort, facilitating swifter and more precise lesion identification, which is essential for timely management in conditions such as Crohn’s disease or gastrointestinal bleeding. Capsules equipped for biopsy and medicine delivery may obviate the need for invasive follow-up operations. Furthermore, magnetically or robotically manipulated capsules may facilitate targeted investigations, enhance diagnostic accuracy, and ensure patient comfort. Collectively, these innovations are poised to enhance diagnostic efficacy, diminish healthcare expenditures, and broaden access to non-invasive gastrointestinal diagnostics.

## Figures and Tables

**Table 1:** The merits and demerits of different types of capsule endoscopes.

Capsule Type	Merits	Demerits
**Steerable**	Precise navigation in upper GI; adjustable view	Larger size; limited battery life; complex control system
**Magnetic**	External control without onboard motor; improved stomach navigation	Limited in lower GI; image distortion; requires external magnetic setup
**Robotic**	Potential for therapeutic tools; high precision	Expensive; complex miniaturization; limited clinical trials
**Tethered**	Real-time control and power; potential for biopsy/intervention	Invasive; patient discomfort; limited to upper GI
**Hybrid**	Combines magnetic and active motion; enhanced mobility	Larger size; integration challenges; high cost
**Conventional**	Small size; commercially approved; safe and widely used	No navigation or therapy; fixed imaging path

## Data Availability

Not applicable.
